# Parotid Hemangioma: A Report of Two Cases

**DOI:** 10.7759/cureus.110825

**Published:** 2026-06-14

**Authors:** Lamya Eliaziji, Ilham Elouardighi, Leila Kartout, Amina Barkat

**Affiliations:** 1 Research Team on Health and Nutrition of Mother and Child, Faculty of Medicine and Pharmacy, Mohammed V University in Rabat, Rabat, MAR; 2 National Reference Center for Neonatology and Nutrition, Centre Hospitalier Ibn Sina, Rabat, MAR; 3 Essaouira Provincial Hospital, Mohammed V Souissi University, Rabat, MAR

**Keywords:** cervical doppler ultrasound, parotid hemangiomas, parotid region, propanolol therapy, vascular tumor parotid gland

## Abstract

Parotid hemangiomas in newborns and infants are rare vascular tumors. Their diagnosis remains difficult and often complex because of the rarity of this condition and the variety of differential diagnoses, such as parotitis or benign parotid tumors. This diagnostic uncertainty can lead to delays in management. Imaging plays a crucial role in confirming the diagnosis.

This case report presents two cases. In the first case, the appearance of the overlying skin and the presence of a dorsal hemangiomatous lesion suggested the diagnosis. The second case presented no diagnostic signs. In both cases, imaging allowed the diagnosis to be established, and treatment with propranolol was initiated with a favorable outcome.

The diagnosis of parotid hemangiomas in infants is a crucial step. The approach is based on a combination of clinical criteria, imaging, and, in rare cases, histopathological analysis, in order to determine the most appropriate treatment as soon as possible.

## Introduction

Parotid hemangiomas in newborns and infants are rare vascular tumors that account for approximately 0.4-0.6% of parotid gland tumors [1]. Diagnosis remains a delicate and often complex process due to the rarity of the condition and the diversity of differential diagnoses, such as parotitis or benign parotid tumors [1,2]. This confusion can lead to delays in treatment. The typical presentation is a painless swelling in the parotid region that develops gradually; imaging plays a key role in confirming the diagnosis [3]. Propranolol is an effective and widely used medical treatment and has been shown to effectively reduce the size of parotid hemangiomas in infants, while being well tolerated and having few side effects. The prognosis remains favorable in the majority of cases [2].

## Case presentation

Case 1

**Figure 1:**
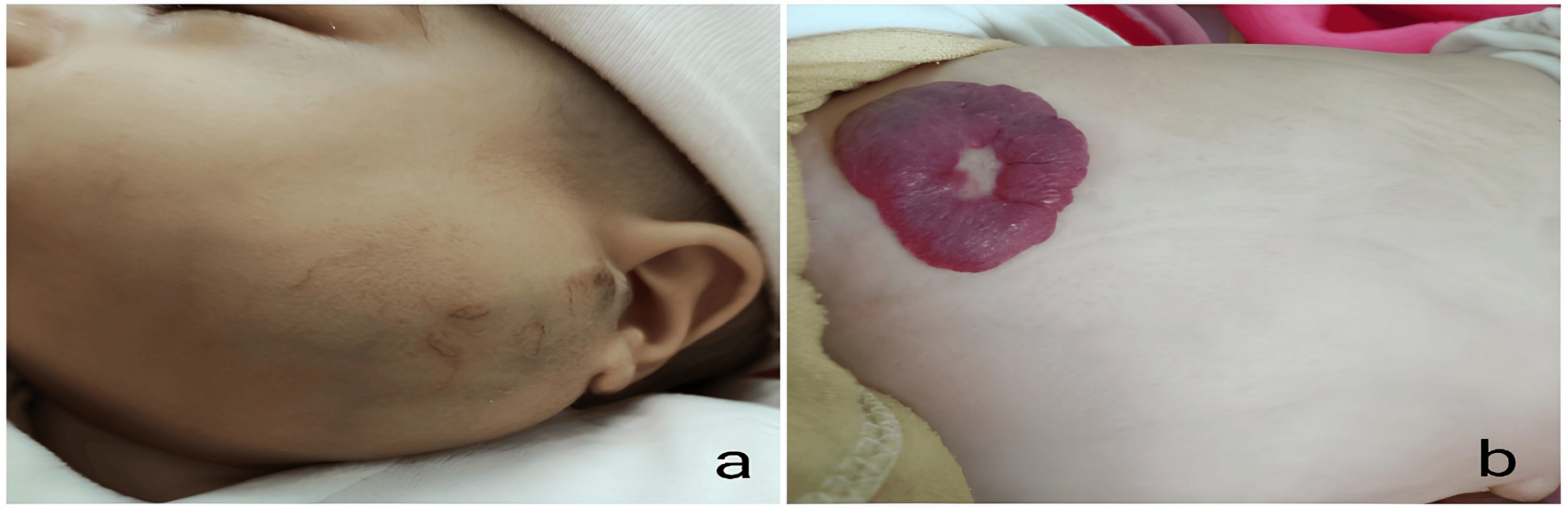
(a) Left parotid mass. (b) Cutaneous hemangioma.

A five-month-old female infant presented with a firm, elastic swelling in the left parotid region that had been present since the age of two months and had gradually increased in size (8 cm × 5 cm), with skin discoloration suggestive of a hemangioma. She also had a raised, reddish-purple skin lesion on her back (Figure 1). 

Cervical Doppler Ultrasound

Enlargement of the parotid gland with a heterogeneous, highly vascularized echotexture and dual arterial and venous components.

Cervical MRI

**Figure 2:**
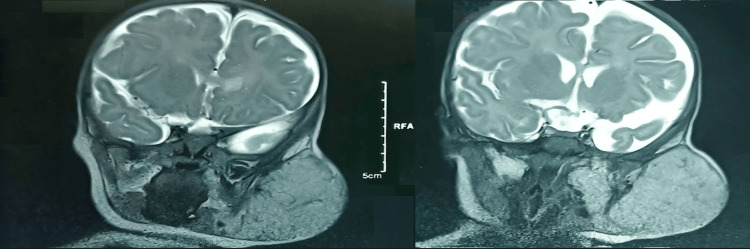
Cervical MRI showing a parotid hemangioma.

A well-defined, oval-shaped lesion involving the left parotid gland, appearing as a T1-hypointense, T2-hyperintense area containing signal-deficient structures that progressively enhance after contrast injection and measuring 55 × 60 × 57 mm. Topographically, it is supplied by the superficial temporal artery. Posteriorly, it comes into contact with the sternocleidomastoid muscle without signs of invasion; anteriorly, it displaces the buccinator muscles; medially, it displaces the pharyngolaryngeal tract and comes into contact with the submandibular gland, with a persistent demarcation line; and laterally, it infiltrates the subcutaneous soft tissues (Figure 2). 

**Figure 3:**
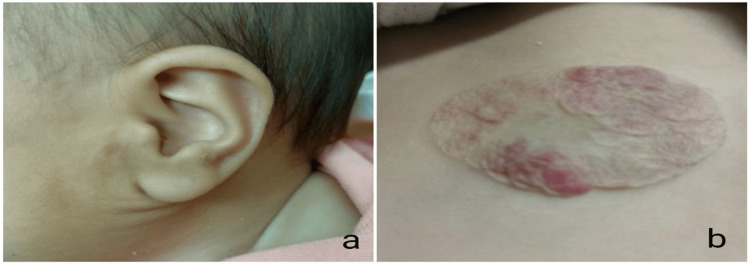
Progression of the hemangioma. (a) Parotid. (b) Cutaneous.

Treatment consisted of propranolol following normal ECG and echocardiography results. The treatment lasted 16 months, and cervical symmetry was achieved within four months of treatment (Figure 3). The patient has been followed up for 42 months with no recurrence. 

Case 2

**Figure 4:**
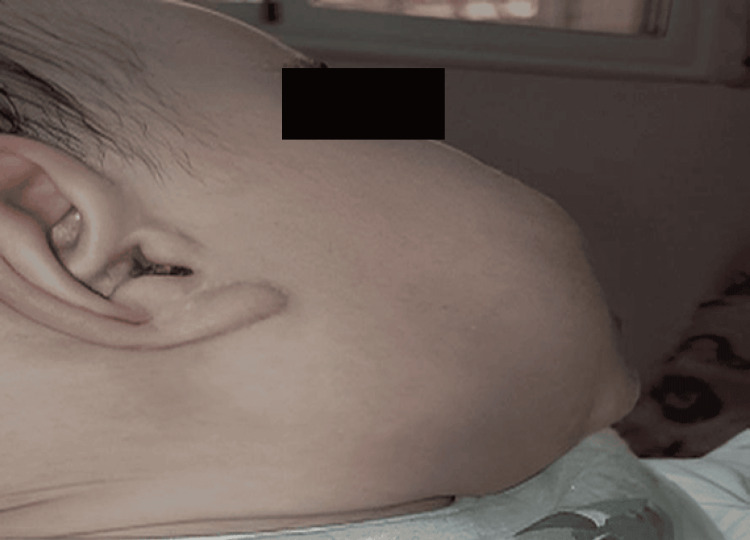
Right parotid mass.

A six-month-old male infant presented with a right parotid cervical mass that had been present since the age of two months and had gradually increased in size to 3-4 cm (Figure 4), with no associated skin findings. 

**Figure 5:**
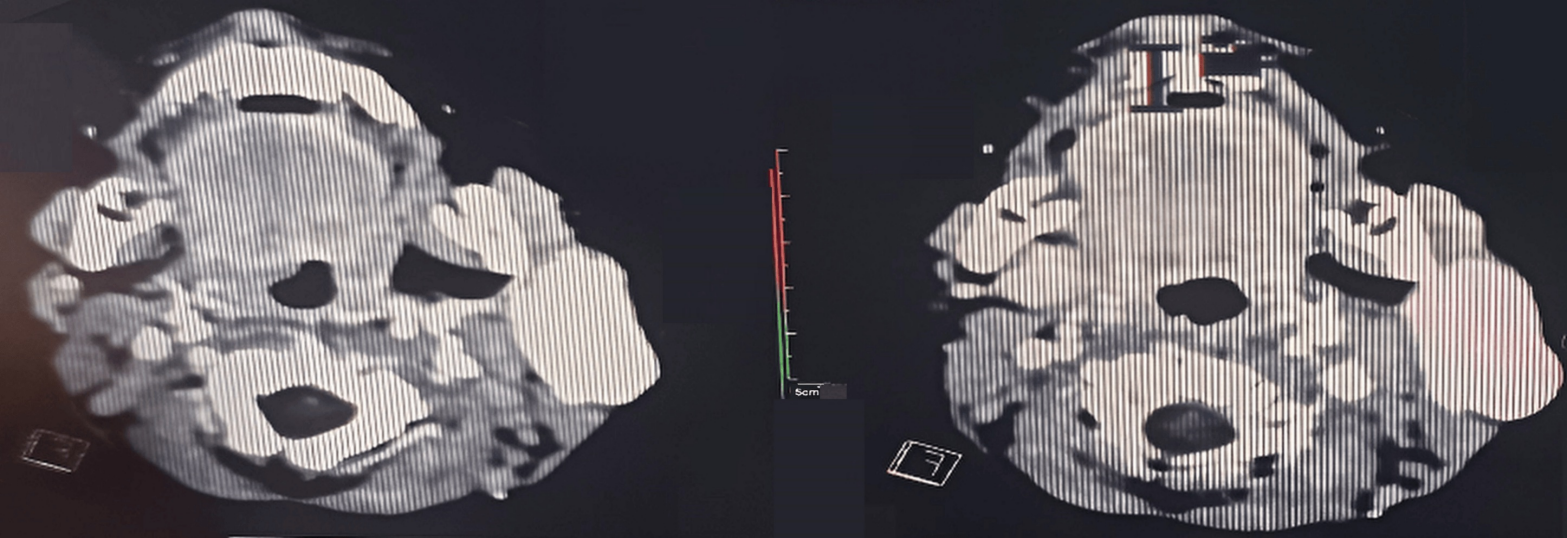
Cervical CT scan showing a hypervascular mass initially suggestive of a hemangioma.

The cervical Doppler ultrasound revealed a large right parotid lymph node enlargement, and cervical CT showed a well-defined, oval-shaped mass with focally lobulated margins, isodense on non-contrast imaging without calcifications, and demonstrating intense, homogeneous enhancement after contrast injection, measuring 33 × 19 × 34 mm. Topographically, posteriorly, it is separated from the right sternocleidomastoid muscle by a fatty plane and comes into contact with the external jugular vein, which remains patent; laterally, it comes into contact with the ipsilateral sublingual gland, with a preserved separating plane; and anteriorly, it comes into contact with the facial vein, also with a preserved separating plane (Figure 5). 

The patient was treated with propranolol for 12 months, with a favorable clinical course and marked improvement after two months. The patient has been followed up for 26 months with no recurrence.

## Discussion

The diagnosis of parotid hemangiomas in infants is a difficult and complex process due to the rarity of the condition and the wide range of differential diagnoses [1,2].

Clinically, the typical presentation is a painless swelling in the parotid region that develops gradually, sometimes accompanied by skin discoloration suggestive of a hemangioma [1]. Initial confusion with acute parotitis or other tumors often delays appropriate management [1,2]. In our first case, the appearance of the skin overlying the lesion and the association with another dorsal lesion helped guide the diagnosis, whereas in the second case, the swelling had no distinctive features.

Imaging plays a fundamental role. Ultrasound allows visualization of a hyperechoic, well-defined mass, suggesting a vascular origin [1]. In cases of doubt, computed tomography (CT) and, especially, magnetic resonance imaging (MRI) provide better tissue characterization, clarify the extent of the lesion and its relationship to adjacent structures, and rule out deep or infiltrating involvement [4]. MRI is particularly indicated for differentiating hemangioma from other benign lesions such as angiomyxoma or malignant lesions and for planning surgical intervention in exceptional cases [2], including biopsy or surgical exploration for histological confirmation following failure of medical treatment or persistent diagnostic uncertainty.

For our patients, Doppler ultrasound combined with MRI in the first case and CT in the second allowed us to rule out differential diagnoses and guide diagnosis and management.

Imaging also plays a role in therapeutic follow-up, particularly in assessing the response to propranolol [3].

Therapeutic approaches remain dominated by propranolol, which is an effective and widely used medical treatment for reducing the size of parotid hemangiomas in infants while being well tolerated and associated with few side effects [2]. Treatment protocols recommend a treatment duration of nine to 12 months and prolonged follow-up to monitor for recurrence [5,6].

Surgery remains the treatment of choice for atypical or large cases or when the diagnosis is confirmed only after surgical exploration [3-7].

Post-treatment follow-up includes clinical evaluation and regular monitoring via ultrasound or MRI [8,9]. Parotid hemangioma remains a benign condition with a favorable prognosis, but certain complications may arise [10,11].

Rapid tumor growth can lead to cosmetic issues and, in cases of significant vascular shunting, signs of congestive heart failure. In addition, obstruction or compression of adjacent structures (such as the external auditory meatus), as well as ulceration of the hemangioma, can lead to serious complications.

Premature surgical exploration can lead to significant complications, such as facial paralysis and functional asymmetry. In cases of propranolol resistance, significant tumor growth may occur, requiring alternatives such as surgery and sclerotherapy [12].

The recurrence rate ranges from 12.6% to 32.9%, depending on the study [13,14]. Recurrence has been associated with several factors, particularly the presence of congenital heart disease, a treatment duration of less than 12 months, and female sex [5,6,15]. In our two cases, there was no associated heart disease; the duration of propranolol treatment was 16 months for the first patient and 12 months for the second, and no recurrence was observed after follow-up periods of 42 months and 26 months, respectively.

The risk of recurrence in infants depends on several clinical, anatomical, and therapeutic factors [8,10,11]. A personalized approach and prolonged treatment duration help limit this risk, but recurrence remains associated with multiple, segmental, or congenital heart disease-associated forms [8-12].

## Conclusions

The diagnosis of parotid hemangiomas in infants is a critical process given the rarity of the condition and the wide range of differential diagnoses. Recognizing the condition and establishing a diagnostic approach require a combination of clinical criteria, imaging, and, in rare cases, histopathological analysis to determine the most appropriate treatment as quickly as possible. Post-treatment follow-up is also critical, as it must be personalized and prolonged given the risk of recurrence and the challenges associated with managing recurrences. Future studies should aim to standardize management and refine the criteria for post-treatment follow-up.
